# Correlation between the Cognitive Status (SIRT1) and the Metabolic Function in Geriatric Patients Using the Indonesian Version of the Montreal Cognitive Assessment (MoCA-INA)

**DOI:** 10.3390/geriatrics8060119

**Published:** 2023-12-05

**Authors:** Made Putra Semadhi, Dewi Muliaty, Eli Halimah, Jutti Levita

**Affiliations:** 1 Prodia National Reference Laboratory, Jakarta 10430, Indonesia; putra.semadhi@gmail.com; 2 Doctoral Program in Pharmacy, Faculty of Pharmacy, Padjadjaran University, Sumedang 45363, Indonesia; 3 Prodia Widyahusada Tbk., Jakarta 10430, Indonesia; dewi.liusvia@prodia.co.id; 4 Department of Pharmacology and Clinical Pharmacy, Faculty of Pharmacy, Padjadjaran University, Sumedang 45363, Indonesia; eli.halimah@unpad.ac.id

**Keywords:** cognitive impairment, glycated hemoglobin, kidney dysfunction, low-density lipoprotein, multimorbidity, Sirtuin 1

## Abstract

A growing life expectancy may result in a chronic medical condition and multimorbidity because the aging process leads to a decrease in cognitive and physiological function. These risks may affect the quality of life of geriatrics. The present study aims to determine the correlation between cognitive status (in terms of SIRT1, a nicotinamide adenine dinucleotide (NAD^+^)-dependent class III deacetylase) and metabolic function (in terms of the lipid profile, kidney function, and blood glucose) in geriatric patients. The differences in the parameters of metabolic function in the participants’ cognitive status were determined by using the Indonesian version of the Montreal Cognitive Assessments (MoCA-Ina). The elderly participants (n = 120) were recruited at three sites in Indonesia from March to October 2022. Our study demonstrated a negative correlation between the cognitive status of geriatric patients and their metabolic function, represented by the MoCA-Ina score with a linear regression equation of *y =* 0.27 − 2.4 ×10^−3^ *x*. Higher levels of LDL-C, cystatin C, and HbA1c were found in the Severe-Moderate Cognitive Impairment group. Determining the SIRT1 levels may be beneficial in predicting both the cognitive and metabolic status of geriatrics because this protein is among numerous metabolic sensors in the hypothalamus.

## 1. Introduction

It was reported in 2010 that the total number of elderly persons (aged > 60 years old) had reached 18 million accounting for 7.6% of Indonesia’s total population. The census has statistically enumerated that the geriatric population will continuously escalate to reach a value of 15.8% of the total population by 2035 [1]. The WHO has estimated the global mean age of life expectancy is 72 years. As the incidence of chronic medical conditions grows with age, multimorbidity is also predicted to elevate. Older persons frequently encounter multimorbidity because the aging process leads to a decrease in cognitive and physiological functions [2,3,4,5]. Of these conditions, mild cognitive impairment (MCI) is considered as one that affects the quality of life.

MCI is defined as cognitive decline greater than expected but that does not significantly interfere with daily activities. It is an intermediate clinical state between normal cognition and dementia and is often used to describe a condition between cognitive age changes and early stages of dementia. According to epidemiological studies, the incidence of MCI is 3–19% in geriatrics [6,7,8]. The criteria comprise memory deprivation, target memory detriment, general cognition preserved, preserved daily activities, and absence of dementia [9]. The American Academy of Neurology described MCI as starting at the age of 60–64 years old and its condition correlates with the health-related quality of life of the patient [10]. However, regardless of the increasing prevalence of MCI, studies in this field have been limited.

The metabolic function of patients strongly influences tissue homeostasis. Specific areas in the hypothalamus record the metabolic information and modulate certain mechanisms for energy homeostasis. Among the metabolic sensors in the brain is Sirtuin 1 (SIRT1). SIRT1 is a nicotinamide adenine dinucleotide (NAD^+^)-dependent class III deacetylase, functioning as a cellular energy sensor and is associated with successful aging, metabolism, cellular differentiation, and apoptosis. The dysregulation of SIRT1 activity will lead to the dysfunction of the brain and neurodegeneration. SIRT1 stimulates lipolysis and reduces fat storage, thus protecting against obesity-induced inflammation [11]. SIRT1 is encoded by the *SIRT1* gene and its role is a target for histone and non-histone proteins. Upon activation, the modulation of its downstream pathways occurs by targeting numerous proteins, i.e., nuclear factor kappa-light-chain-enhancer of activated B cells (NF-kappaB), peroxisome proliferators-activated receptor-gamma (PPAR-γ), and many others [12].

Taking everything into consideration, our study aims to determine the correlation between cognitive status (in terms of SIRT1) and metabolic function (in terms of the lipid profile, kidney function, and blood glucose) in geriatric patients. The differences in the parameters of metabolic function in the cognitive status of the participants were determined by using the Indonesian version of the Montreal Cognitive Assessments (MoCA-Ina). The MoCA is used to detect mild cognitive dysfunctions in any condition, including Alzheimer’s disease, vascular cognitive impairment, Parkinson’s disease, and other various neurologic disorders by appraising numerous cognitive domains such as executive and visuospatial function, attention and concentration, memory, language, calculation, and orientation [13,14,15]. MoCA is a sensitive screening instrument and has been reported to successfully determine the scores between male schizophrenia prescribed by risperidone and adjunctive donepezil in a public hospital in Indonesia [16].

## 2. Materials and Methods

### 2.1. Ethics Approval and Informed Consent

The study was conducted following the latest version of the Declaration of Helsinki and approved by the Research Ethics Committee of Universitas Padjadjaran Bandung, Indonesia, with the approval document number 393/UN6.KEP/EC/2021. The geriatric participants signed the informed consent and agreed to their data being published.

### 2.2. Study Design and Population

Following written informed consent, a cross-sectional study was conducted on 120 geriatric participants (age > 60 years old; male 60 participants and female 60 participants). The participants were recruited at three sites in Indonesia (Jakarta, Bogor, and Denpasar) from March to October 2022. All participants were interviewed and clinically examined for their health status at the Prodia Clinical Laboratory and participants with T2DM, an acute or chronic condition of disorders, cancer, chronic kidney disorders, liver dysfunction, and/or chronic obstructive pulmonary disease were excluded.

### 2.3. Assessment of Clinical Chemistry Parameters

Venous blood (median cubital vein) was taken from the arm of the participants by a clinician. The blood sample was assayed for HbA1c, cystatin C, and low-density lipoprotein-cholesterol (LDL-C), following the standard operational procedure of the Prodia Clinical Laboratory (https://prodia.co.id/id/index; accessed on 22 October 2023) as described below:

#### 2.3.1. Glycated Hemoglobin (HbA1c)

HbA1c in whole blood collected in an ethylenediaminetetraacetic acid K3 tube was reacted with HbA1C kit-2.0 reagent (catalog no. 12000447, Bio-Rad, Hercules, CA, USA) by using an immunoassay ion-exchange high-performance liquid chromatography (HPLC) system (Variant II Turbo, Bio-Rad).

#### 2.3.2. Cystatin C

The whole blood was collected in a serum separator tube (SST), and centrifugated at 3000 rotations per minute for 10 min, and cystatin C in the serum was reacted with N Latex Cystatin C reagent assay (catalog no. OQNM, Siemens, Erlangen, Germany) using the nephelometric method (the BN ProSpec System, Siemens). The glomerular filtration rate (GFR) was calculated using the chronic kidney disease epidemiology collaboration (CKD-EPI) equation.

#### 2.3.3. Low-Density Lipoprotein-Cholesterol (LDL-C)

The whole blood was collected in a serum separator tube (SST), and centrifugated at 3000 rotations per minute for 10 min, and LDL-C in the serum was determined using the enzymatic method (Architect C System, Abbott, IL, USA) with a reagent R1 Kit for Cholesterol oxidase, Cholesterol esterase, Peroxidase, 4-amino antipyrin, and HBA (catalog no. 7D62).

### 2.4. Assessment of the Cognitive Status (MCI) Biomarker (SIRT1)

The whole blood was collected in a serum separator tube (SST), incubated for 2 h at 4 °C, cold-centrifugated at 1000 g for 20 min at 4 °C, and SIRT1 in the serum was reacted with Human SIRT1 (catalog no. EKX-6ZK077-96, Nordic Biosite AB, Taby, Sweden) using the ELISA method (Microplate Reader model 680, Bio-Rad Lab, Hercules, CA, USA).

### 2.5. Statistical Analysis

The IBM Statistical Package for the Social Sciences (SPSS) v.22 for Microsoft Windows was used to assess the distribution pattern of the data, by employing the Shapiro-Wilk test, followed by Pearson’s correlation, *t*-test, and one-way ANOVA test.

## 3. Results

### 3.1. Characteristics of the Participants

The data in Table 1 confirm that, in general, the participants tend to possess a good health status as their clinical chemistry and cognitive biomarkers are within the normal range with an average MoCA-Ina score of 22 for males and 20 for females. On average, both male and female participants are categorized as having mild cognitive impairment with a homogeneity between the two groups, however, there is a significant difference in the SIRT1 levels (*p* < 0.05). In all parameters evaluated, the female participants exhibited a lower value compared to those of the males, although the age and body weight of the females were higher, which were 68 years old (maximum 86 years old and minimum 60 years old) vs. 67 years old (maximum 87 years old and minimum 62 years old) and 63.3 (maximum 76 and minimum 43) vs. 51.4 (maximum 73 and minimum 44), respectively. The blood glucose levels of the females were slightly higher than the males, as shown by the HbA1c values.

Based on the data in Table 1, we further assessed the correlation between the clinical biomarkers and the cognitive status of the male and female participants; therefore, the resulting data were classified according to the MoCA-Ina score.

### 3.2. Performance of the MoCA-Ina and Association with Demographic and Clinical Variables

Twenty participants were categorized into the Severe-Moderate Cognitive Impairment group (MoCA-Ina score ≤ 17), seventy-six participants were in the Mild Cognitive Impairment group (MoCA-Ina score 18–25), and twenty-four participants were in the Normal Cognitive group (MoCA-Ina score ≥ 26). Interestingly, all biomarkers revealed notable differences between groups (*p* < 0.05). The better the cognitive status, the lower the levels of HbA1c, LDL-C, cystatin C, and SIRT1. These data imply that geriatric participants with Severe-Moderate Cognitive Impairment indicated a higher level of HbA1c, cystatin C, and SIRT1 compared to those of the other two groups, regardless of their genders. The results are tabulated in Table 2.

Furthermore, a statistical correlation between the clinical biomarkers and MoCA-Ina scoring is presented in Table 3, the correlation between the clinical biomarkers and the SIRT1 levels is tabulated in Table 4, and the correlation between clinical biomarkers and ages is described in Table 5. In the latter, the gender of the participants was omitted.

The MoCA-Ina score of each category against the clinical biomarkers revealed various results (Table 3); for example, HbA1c NGSP (R 0.256; *p* < 0.05) and cystatin C (R 0.266; *p* < 0.05) demonstrated a significant positive correlation with the MoCA-Ina score, whereas the SIRT1 levels demonstrated a significant negative correlation in the Severe-Moderate Cognitive Impairment group (R −0.502; *p* < 0.05). Moreover, in the Mild Cognitive Impairment group, the levels of both HbA1c NGSP and IFCC (R −0.224; *p* < 0.05) and cystatin C (R 0.296; *p* < 0.05) exhibited a significant negative correlation against the MoCA-Ina score, whereas the SIRT1 levels showed a significant positive correlation against the MoCA-Ina score in participants with Normal Cognitive (R 0.437; *p* <0.05).

**Figure 1:**
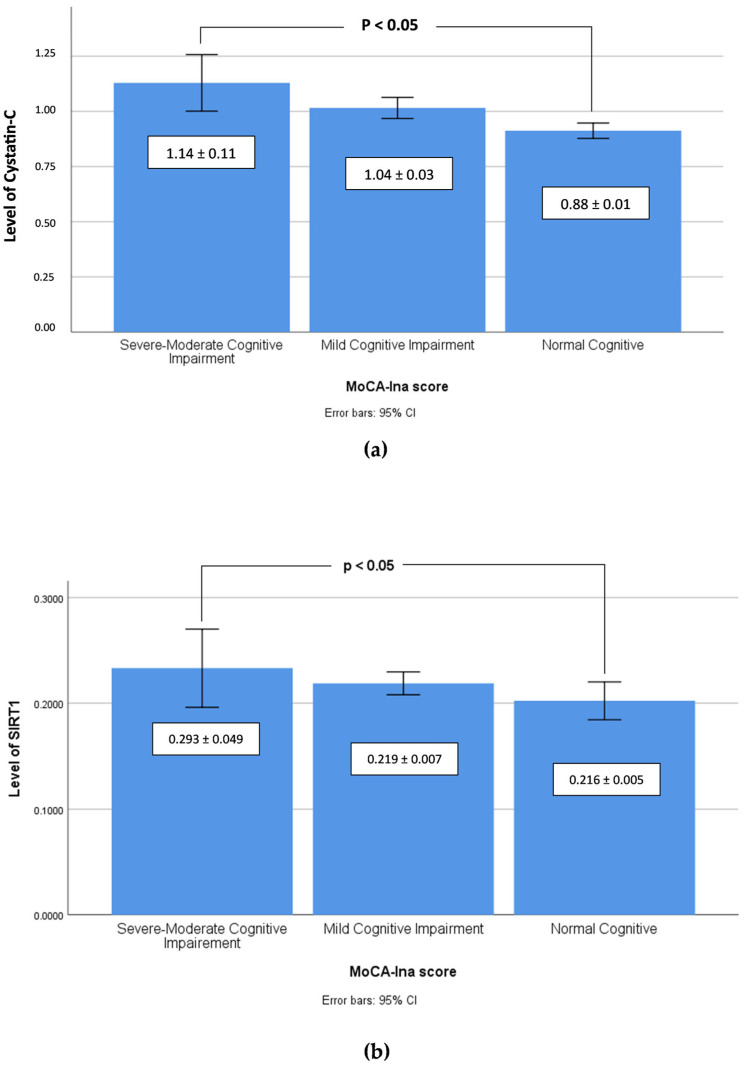
Column chart of the MoCA-Ina score (95% CI) using the Pearson’s correlation test reveals the differences between the mean levels of (**a**) cystatin C and (**b**) SIRT1 in Severe-Moderate Cognitive Impairment, Mild Cognitive Impairment, and Normal Cognitive groups.

**Figure 2:**
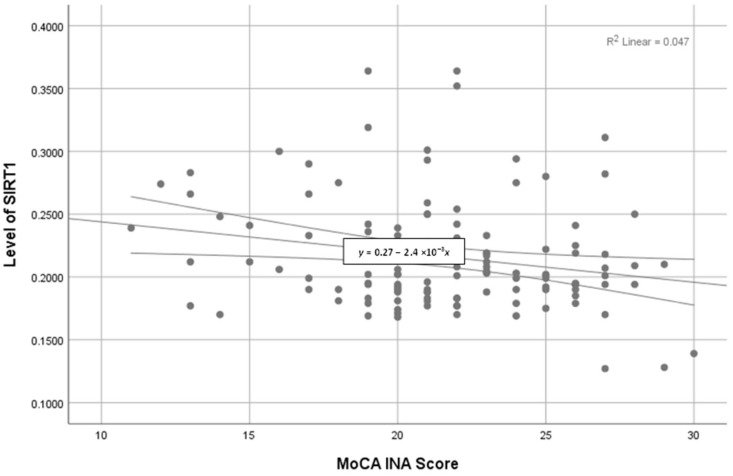
Scatter chart of SIRT1 levels against the MoCA-Ina score using the Pearson test reveals a negative correlation (linear regression equation *y =* 0.27 − 2.4 × 10^−3^ *x*).

The correlation between clinical biomarkers and the SIRT1 levels presented in Table 4 reveals that a significant negative correlation was observed in the Severe-Moderate Cognitive Impairment group (*p* < 0.05), while a significant positive correlation occurred in the Normal Cognitive group (*p* < 0.05). Furthermore, the MoCA-Ina score of the differences between the mean levels of (a) cystatin C and (b) SIRT1 in the three cognitive groups is depicted as a column chart in Figure 1, and the correlation between the MoCA-Ina score and the SIRT1 levels of all participants, regardless their groups, is depicted in Figure 2.

There was no significant correlation between age and clinical biomarkers, as described in Table 5, indicating that no interference from the level of age to the level of biomarkers in the three cognitive groups.

## 4. Discussion

The main finding of this study confirms the development of a negative correlation between the cognitive status of geriatrics and their metabolic function, implying that the better the cognitive status, the lower the levels of HbA1c, LDL-C, cystatin C, and SIRT1.

Metabolic status plays a pivotal role in the quality of life of a person, particularly with increasing age. The decline in the metabolic function of geriatrics eventually leads to the imbalance of tissue homeostasis. SIRT1 is among the metabolic sensors in the hypothalamus [11]. The hypothalamus controls the regulation of body temperature, nutrient intake and energy balance, the sleep and wake cycle, and numerous other important processes. Therefore, due to its impact on a multitude of health problems, attention has been focused on the superior role of the hypothalamus in systemic aging [17].

A cohort study consisting of 175 patients with cognitive decline and 335 non-demented controls, conducted by Cho et al. (2015), demonstrated that, in humans, hypomethylation of interleukin-1β is strongly correlated with chronological age and with increased IL-1β transcription [18]. Review studies by Elibol and Kilic (2018) [12] and Fagerli et al. (2022) [19] described that the interaction of healthy aging, age-related diseases, and calorie restriction may contribute to the enhancement of SIRT1 activity. Moreover, a positive correlation between age and SIRT1 activity leads to compensation against oxidative stress. Several initiative conditions of neurodegenerative are marked by the occurrence of neuroinflammation, mitochondrial damage, and an increase in oxidative stress. In these cases, the levels of SIRT1 showed a tendency to elevate [12,20]. Mitochondrial well-being is considered an indicator of neurodegenerative status, because this organelle serves critical roles in controlling reactive oxygen species [21]. A previous study by Kumar et al. (2013) described that the range level of SIRT1 measured from MCI subjects was 3.35 to 3.81 ng/µL, whereas that from AD subjects was 1.37 to 2.99 ng/µL, compared to the normal elderly group, 4.41 to 5.96 ng/µL. The SIRT1 levels in this study were measured by employing ELISA technology [22]. Similarly, a study by Le Couteur and coworkers (2011) reported that serum-induced SIRT1 expression was not different between frail and robust male participants aged more than 70 years old (103.1 ± 17.0 vs. 100.4 ± 19.3 mg/L) [23]. It was described that the SIRT1 protein levels were remarkably higher in elderly Turkish patients (4.07 ± 0.22 ng/mL) compared to that of pediatric patients (1.58 ± 0.07 ng/mL) and adults (1.84 ± 0.10 ng/mL) (*p* < 0.001) [24].

Becoming older is frequently related to the diminishing of the functions of important organs, cognitive function is among those. The present study demonstrated the significant differences in HbA1c levels between groups and the significant correlation between the MoCA-Ina score in the Severe-Moderate Cognitive Impairment and the Mild Cognitive Impairment groups. This infers the controlling effect of blood glucose metabolism on the neurocognitive of an elderly person. The better the HbA1c value, the better the cognitive status of the geriatrics, and vice versa.

Our data are in accordance with a previous cross-sectional study by Binder and co-workers (2017) conducted in Germany with 113 patients (70 females and 43 males, ages ranging between 50–87 years old). It was confirmed that the HbA1c value was inversely correlated with various measures of cognitive condition; thus, high levels of blood glucose were verified as a major risk factor for cognitive impairment [25]. Interestingly, a nationwide, register-based cohort with all diabetes cases conducted by Andersen and co-workers in Denmark from January 2000 through December 2012 (n > 150,000) revealed that high HbA1c levels were correlated with lessened cognitive performance [26]. Another study of 311 patients (aged 23 to 96 years old) with acute mild ischemic stroke at the Suining Central Hospital, Sichuan Province, China, from 1 January 2015 to 31 December 2018, demonstrated almost similar results. This study, carried out by Xu and the research team, noted a nonlinear relationship between HbA1c and post-stroke cognitive impairment. It was described that when the HbA1c value exceeded 8.2%, it was positively correlated with post-stroke cognitive impairment [27]. Glycosylated or glycated hemoglobin A1c (HbA1c) is deemed to be the biomarker for the three months of average blood glucose levels. It has been confirmed that the normal HbA1c level should not exceed 6.5% [28]. HbA1c is the product of the combination of hemoglobin and blood sugar. Its levels in the blood are stable and are not influenced by short-term blood glucose levels. Currently, HbA1c is globally accepted as the gold standard for chronic blood glucose control [29,30]. Nonetheless, Chen and co-workers (2023) advocated that the recommended HbA1c by ADA criterion may not be sufficiently sensitive to diagnose high blood glucose levels in patients with pancreatic dysfunction. For these patients, a combination of HbA1c and fasting plasma glucose (FPG) assessments should be employed. In their cross-sectional study conducted at the West China Hospital, Chen and co-workers assessed 732 participants, comprising 331 as the control group and 401 participants diagnosed with pancreatic dysfunction [31]. A previous study reported that the correlation between glucose levels and HbA1c is not the same in the Inuit ethnicity (unique indigenous peoples of the Arctic and sub-Arctic regions) and Danish participants, thus, HbA1c may not be accurate in diagnosing diabetes [32].

The geriatric patients recruited in the present study denoted a high level of LDL-C (>120 mg/dL), which significantly differed between groups and gradually decreased from severe to normal. The normal group showed the lowest LDL-C level. However, no significant correlation was found between the MoCA-Ina score and the LDL-C levels in each group. A potential negative correlation was observed between these variables in the Normal Cognitive group. It could be considered that the maintained levels of LDL-C are associated with better cognitive performance to slow the potential of cognitive decline.

In comparison with our study, a community-based longitudinal study of Chinese elderly (n = 1,159; females 48.7%) conducted by Ma and co-workers in China from 1998 to 2014 indicated that higher levels of total cholesterol and LDL-C were linked with a rapid deterioration in the Chinese Mini-Mental State Examination (MMSE) score over time [33].

A high concentration of cholesterol contributes to the buildup of amyloid beta peptides, which increases the occurrence of cognitive impairment. It was proven that cholesterol levels modulated the processing of the amyloid precursor protein (APP) in animal models [34]. Another study of 7129 participants (2832 males and 4297 females) conducted in Wuhan, China, delineated that low LDL-C levels (<70 mg/dL, especially <55 mg/dL) were correlated with notable slower cognitive decline in a population-based setting [35]. Similarly, a population-based cohort study conducted in Dijon, Bordeaux, and Montpellier, France, between March 1999 and March 2001 revealed that higher LDL-C and total cholesterol levels were correlated with an elevated risk of Alzheimer’s disease [36].

In our study, the cystatin C levels of the Severe-Moderate Cognitive Impairment group were higher compared to the Mild Cognitive Impairment and the Normal Cognitive groups. Our results are in accordance with a previous study carried out by Zhang and colleagues (2021) in China. In their study, a multiple linear regression model was employed to evaluate the correlation between cystatin C levels and the cognitive status of 117 patients with multiple system atrophy (MSA) compared to 416 healthy participants and it showed that cystatin C levels were significantly higher in patients with MSA. Cystatin C levels were negatively correlated with the MoCA score [37]. A study of 463 patients with Alzheimer’s disease conducted in China indicated that higher serum cystatin C may be linked with worse cognitive performance. This study confirmed the involvement of cystatin C in the pathogenesis of Alzheimer’s disease (AD) and cognitive impairment [38].

Cystatin C is synthesized at a constant rate and is freely filtered, reabsorbed, and degraded in the proximal tubule of the nephron; therefore, it is used as the marker for assessing the filtration function of the kidney [39]. The diagnostic role of cystatin C as an indicator of kidney damage has been broadly studied in humans. In these clinical studies, cystatin C proved to have better diagnostic accuracy compared to serum creatinine in differentiating a normal kidney function from an injured one [40]. Cross-sectional data taken from 18,253 participants aged 28–100 years old revealed that there may be a strong, non-linear correlation between age and the function of the kidneys, by measuring the cystatin C levels, even in healthy persons. The levels of cystatin C substantially increased with age, even in the absence of clinical risk factors for kidney disorders [41]. A study of African-American participants with hypertension (n = 1094), aged 18–70 years old, with a GFR between 20–65 mL/minute/1.73 m^2^, demonstrated that in accordance with the decrease in kidney function, there may be elevated stress on the remaining nephrons [42].

Our cross-sectional study was conducted on 120 elderly participants (age > 60 years old; male 60 participants and female 60 participants), this small sample size may be one of the limitations of this study. Usually for studies on geriatric patients, a longitudinal study design is preferred, however, many researchers have shown a preference for cross-sectional designs [41,43,44,45]. It was outlined from a cross-sectional study conducted in China (n = 425) that cognitive frailty was predominant among geriatric patients with chronic kidney disease, and early prediction and intervention may avert the incidence of this ailment [45].

## 5. Conclusions

The present study was conducted on 120 geriatric patients recruited in three big cities in Indonesia. In this study, we confirmed that there is a negative correlation between the cognitive status (in terms of SIRT1) of geriatrics and their metabolic function (in terms of the LCD-C, cystatin C, and HbA1c levels). Higher levels of LDL-C, cystatin C, and HbA1c were found in the Severe-Moderate Cognitive Impairment group compared to the Mild Cognitive Impairment and the Normal Cognitive groups. Determining the SIRT1 levels may be beneficial in predicting both the cognitive and metabolic status of geriatrics because this particular protein is among numerous metabolic sensors in the hypothalamus. The limitation of this study is the sample size (n = 120), because the larger the sample size, the better the accuracy of the results. Moreover, other biomarkers are still needed to predict the decline of neurological status or cognitive conditions in elderly patients, thus opening the chance for further explorations.

## Figures and Tables

**Table 1:** Demographic and Health Characteristics of the Participants.

Demographics	Male	Female	*p*
Age (years)	67	68	0.025
Body weight (kg)	51.4	63.3	0.378
**Clinical chemistry parameter**			
HbA1c NGSP (%)	5.7 ± 0.9	5.9 ± 0.1	0.790
HbA1c IFCC (mmol/mol)	39.5 ± 1.0	41.3 ± 1.3	0.882
LDL-C (mg/dL)	140.4 ± 4.8	137.8 ± 4.2	0.258
Cystatin C (mg/dL)	1.04 ± 0.03	0.99 ± 0.02	0.326
GFR Creatinine-Cystatin (mL/min/1.73 m^2^)	76.2 ± 2.1	80.1 ± 2.0	0.252
**Cognitive status biomarker**			
SIRT1 (ng/mL)	0.243 ± 0.005	0.230 ± 0.018	<0.05 **
**Cognitive Assessment result**			
MoCA-Ina Score	22	20	0.463

Data are presented as mean ± SEM; HbA1c NGSP: the value of glycated hemoglobin calculated according to the National Glycohemoglobin Standardization Protocol; HbA1c IFCC: the value of glycated hemoglobin calculated according to the International Federation of Clinical Chemistry. Statistical analysis was performed using the student *t*-test. ** indicates a significant difference.

**Table 2:** MoCA-Ina Scoring of the Participants Resulted in Three Categories: Severe-Moderate Cognitive Impairment, Mild Cognitive Impairment, and Normal Cognitive.

	MoCA-Ina Scoring						
	Severe-Moderate Cognitive Impairment		Mild Cognitive Impairment		Normal Cognitive		
Demographics	MoCA-Ina Score ≤17 (N = 20)		MoCA-Ina Score 18–25 (N = 76)		MoCA-Ina Score ≥ 26 (N = 24)		*p*
	Male	Female	Male	Female	Male	Female	
Age (years)	66	70	67	68	65	66	0.692
Body weight (kg)	64	55	66	61	66	63	0.513
**Clinical chemistry parameter**							
HbA1c NGSP (%)	6.1 ± 0.5	6.1 ± 0.4	5.8 ± 0.1	5.9 ± 0.1	5.4 ± 0.2	5.4 ± 0.3	<0.05 **
HbA1c IFCC (mmol/mol)	42.8 ± 5.1	42.8 ± 3.8	40.2 ± 1.2	40.9 ± 1.5	35.9 ± 1.7	36.3 ± 3.0	<0.05 **
LDL-C (mg/dL)	155.0 ± 19.5	142.8 ± 8.0	139.7 ± 5.7	138.6 ± 6.0	135.1 ± 9.0	128 ± 7.8	<0.05 **
Cystatin C (mg/dL)	1.2 ± 0.1	1.1 ± 0.1	1.1 ± 0.0	1.0 ± 0.0	0.9 ± 0.0	0.9 ± 0.0	<0.05 **
GFR Creatinine-Cystatin (mL/min/1.73 m^2^)	65.5 ± 7.3	72.5 ± 5.7	74.8 ± 2.6	81.4 ± 2.4	86.1 ± 3.0	85.3 ± 3.0	<0.05 **
**Cognitive status biomarker**							
SIRT1 (ng/mL)	0.225 ± 0.015	0.338 ± 0.793	0.215 ± 0.006	0.223 ± 0.009	0.213 ± 0.003	0.205 ± 0.006	<0.05 **

HbA1c NGSP: the value of glycated hemoglobin calculated according to the National Glycohemoglobin Standardization Protocol; HbA1c IFCC: the value of glycated hemoglobin calculated according to the International Federation of Clinical Chemistry. Statistical analysis was performed using the one-way ANOVA test. ** indicates a significant difference.

**Table 3:** Correlation Between Clinical Biomarkers and the MoCA-Ina Scoring for Severe-Moderate Cognitive Impairment, Mild Cognitive Impairment, and Normal Cognitive Group.

	Correlation to MoCA-Ina Scoring					
	Severe-Moderate Cognitive Impairment (N = 20)		Mild Cognitive Impairment (N = 76)		Normal Cognitive (N = 24)	
Clinical Chemistry Parameter	Coefficient Correlation	*p*	Coefficient Correlation	*p*	Coefficient Correlation	*p*
HbA1c NGSP (%)	0.256 *	<0.05 *	−0.224 *	<0.05 *	−0.062	0.773
HbA1c IFCC (mmol/mol)	0.254 *	<0.05 *	−0.224 *	<0.05 *	−0.062	0.773
LDL-C (mg/dL)	0.129	0.588	0.142	0.206	−0.181	0.397
Cystatin C (mg/dL)	0.266 *	<0.05 *	−0.295 *	<0.05 *	−0.018	0.935
GFR Creatinine-Cystatin (ml/min/1.73 m^2^)	−0.787 *	<0.05 *	0.296 *	<0.05 *	0.004	0.985
**Cognitive status biomarker**						
SIRT1 (ng/mL)	−0.502 *	<0.05 *	0.069	0.543	0.437 *	<0.05 *

HbA1c NGSP: the value of glycated hemoglobin calculated according to the National Glycohemoglobin Standardization Protocol; HbA1c IFCC: the value of glycated hemoglobin calculated according to the International Federation of Clinical Chemistry. Statistical analysis was performed using the Pearson test. * indicates a significant correlation.

**Table 4:** Correlation between Clinical Biomarkers and the SIRT1 Levels for Severe-Moderate Cognitive Impairment, Mild Cognitive Impairment, and Normal Cognitive Group.

	Correlation to SIRT1					
	Severe-Moderate Cognitive Impairment (N = 20)		Mild Cognitive Impairment (N = 76)		Normal Cognitive (N = 24)	
Clinical Chemistry Parameter	Coefficient Correlation	*p*	Coefficient Correlation	*p*	Coefficient Correlation	*p*
HbA1c NGSP (%)	−0.216 *	<0.05 *	−0.204 *	<0.05 *	−0.192 *	<0.05 *
HbA1c IFCC (mmol/mol)	−0.224 *	<0.05 *	−0.234 *	<0.05 *	−0.182 *	<0.05 *
LDL-C (mg/dL)	−0.109	0.548	−0.132	0.236	−0.121	0.397
Cystatin C (mg/dL)	0.207 *	<0.05 *	0.275 *	<0.05 *	0.218 *	<0.05 *
GFR Creatinine-Cystatin (mL/min/1.73 m^2^)	−0.187 *	<0.05 *	−0.196 *	<0.05 *	−0.204	<0.05 *
**Cognitive Assessment Result**						
MoCA-Ina score	−0.502 *	<0.05 *	0.069	0.543	0.437 *	<0.05 *

HbA1c NGSP: the value of glycated hemoglobin calculated according to the National Glycohemoglobin Standardization Protocol; HbA1c IFCC: the value of glycated hemoglobin calculated according to the International Federation of Clinical Chemistry. Statistical analysis was performed using the Pearson test. * indicates a significant correlation.

**Table 5:** Correlation between Clinical Biomarkers and the Ages of the Participants.

	Correlation to Age					
	Severe-Moderate Cognitive Impairment (N = 20)		Mild Cognitive Impairment (N = 76)		Normal Cognitive (N = 24)	
Clinical Chemistry Parameter	Coefficient Correlation	*p*	Coefficient Correlation	*p*	Coefficient Correlation	*p*
HbA1c NGSP (%)	0.140	0.556	0.050	0.657	0.292	0.166
HbA1c IFCC (mmol/mol)	0.140	0.556	0.050	0.657	0.277	0.189
LDL-C (mg/dL)	−0.275	0.240	−0.010	0.927	−0.018	0.933
Cystatin C (mg/dL)	0.040	0.867	0.144	0.202	−0.065	0.764
GFR Creatinine-Cystatin (mL/min/1.73 m^2^)	−0.032	0.892	−0.120	0.288	0.031	0.884
**Cognitive Assessment Result**						
SIRT1 (ng/mL)	−0.029	0.549	−0.002	0.987	−0.268	0.205
MoCA-Ina score	−0.383	0.305	−0.064	0.571	−0.186	0.385

HbA1c NGSP: the value of glycated hemoglobin calculated according to the National Glycohemoglobin Standardization Protocol; HbA1c IFCC: the value of glycated hemoglobin calculated according to the International Federation of Clinical Chemistry. Statistical analysis was performed using the Pearson test. There was no significant correlation indicated.

## Data Availability

Data is available upon request to the first author.
